# Prevalence and clinical profile of insulin resistance in young women of poly cystic ovary syndrome: A study from Pakistan

**DOI:** 10.12669/pjms.292.3180

**Published:** 2013-04

**Authors:** Rumina Tabassum, Fouzia Imtiaz, Shaheen Sharafat, Shazia Shukar-ud-din, Uzma Nusrat

**Affiliations:** 1 Dr. Rumina Tabassum, FCPS, Associate Professor of Obs & Gynae, Dow University of Health Sciences (DUHS), Karachi, Pakistan.; 2 Dr. Fouzia Imtiaz, PhD, Associate Professor of Biochemistry, Dow University of Health Sciences (DUHS), Karachi, Pakistan.; 3 Dr. Shaheen Sharafat, PhD, Professor of Pathology, Dow University of Health Sciences (DUHS), Karachi, Pakistan.; 4 Dr. Shazia Shukar-ud-din, FCPS, Assistant Professor of Obs & Gynae, Dow University of Health Sciences (DUHS), Karachi, Pakistan.; 5 Dr. Uzma Nusrat, FCPS, Senior Registrar of Obs & Gynae, Dow University of Health Sciences (DUHS), Karachi, Pakistan.

**Keywords:** Poly Cystic Ovary Syndrome, Insulin Resistance, Waist Hip Ratio, Acanthosis Nigrican

## Abstract

***Objective:*** The aim was to estimate the prevalence of Insulin Resistance (IR) in Poly Cystic Ovary Syndrome (PCOS) and analyze its clinical parameters.

***Methodology: ***This observational study was conducted at Dow University Hospital during June 2011 till May 2012. Patients of PCOS were selected, an anthropometric measurement, examination and fasting blood test for sugar (FBS) and insulin was performed. Data was collected on pre designed questionnaire, was analyzed by SPSS version 16.

***Results: ***Forty-six cases of PCOS were included in the study. Prevalence of IR was 34.78%. Mean age of patients was 23.72 ± 4.37 years. Waist Hip Ratio (WHR) was raised in 42 (91.30%), acanthosis was found in 26(56.50%), impaired FBS was seen in 9 (19.6%) and raised fasting insulin in 16 (34.8%) patients. There was significant association between acanthosis and WHR (0.044) and between acanthosis and FBS (0.008). Correlation studies between parameters showed a significant correlation between Waist & Hips (0.93), similarly Waist & WHR showed positive correlation (0.59), at p< 0.01. Significant positive correlation was also found between waist and FBS (0.32) and FBS & WHR (0.378).

***Conclusion: ***Acanthosis nigrican, raised WHR and FBS are significant parameters for insulin resistance in cases of Poly Cystic Ovary Syndrome (PCOS).

## INTRODUCTION

Insulin resistance (IR) is defined clinically as the inability of a known quantity of exogenous or endogenous insulin to increase glucose uptake and utilization in an individual as much as it does in a normal population. Body mass Index (BMI) is highly predictive of Insulin resistance and impaired fasting blood glucose in women with Polycystic Ovary Syndrome (PCOS)^1^: It is a disease of heterogeneous disturbance of reproductive, endocrine and metabolic functions.^2^ PCOS is one of the most common endocrine disorders of women in her reproductive age, its prevalence is 4-12%,^3^ while in some population it may be as high as 25%.^4^

In 2003, the Rotterdam consensus expanded the diagnostic criteria to include at least two of the following three features: clinical and/or biochemical hyperandrogenism, oligoanovulation, and polycystic ovaries, excluding other endocrinopathies.^5^ In Family history about 40- 50% of first degree relatives have POCS, suggesting a dominant mode of inheritance.^6^ Insulin resistance (IR) is present in both obese and non-obese women with PCOS, evidence supports the association between PCOS and region near the Insulin receptor gene.^7^ about 50-70% of women with PCOS demonstrate insulin resistance beyond that predicted by their BMI.^8^ IR causes compensatory hyperinsulinemia, that results into hyperandrogenism by stimulating ovary and increasing free androgen by suppressing liver production of Sex Hormone Binding Globulin.^2^ Approximately 75% patients with PCOS are found to be insulin resistant.^9^ Insulin resistance is reported 40.2% from Czech Republic^10^ and 76.9% from India^11^ in women with PCOS.

Clinical features vary from asymptomatic cases to oligomenorrhea/amenorrhea/irregular periods, subfertility, first trimester miscarriage, Obesity, Hirsutism, Acne^12^, Acanthosis nigricans and male pattern alopecia.^3^ Central obesity and acanthosis nigricans are signs of IR^3 ^in PCOS and also increase the risk of type II diabetes mellitus by 5–10 times.^13^ By the age of forty, 40% of the PCOS patients develop type II diabetes mellitus or impaired glucose tolerance.^14^

The objective of this study was to estimate the prevalence of insulin resistance in polycystic ovary syndrome (PCOS) and to see correlation of clinical parameters of Insulin Resistance in women of reproductive age.

## METHODOLOGY

This was an observational study, conducted in outpatient department of Dow University Hospital from June 2011 till May 2012.

 Diagnosed cases of PCOS were scrutinized for inclusion criteria of women below 35 years of age, non- pregnant and non- lactating, married or single, last abortion or delivery more than three months back, and with no history of diabetes and any other endocrine disorder. Those who were on insulin sensitizing agent within three months were excluded. Informed consent was taken from each selected patient after explaining the study purpose. After taking a thorough history, anthropometric measurement and general physical examination was performed.

Three milliliter of venous blood was collected in the morning after overnight fast (12-14 hours) for fasting blood sugar (FBS) and serum insulin level at Dow Diagnostic Research & Reference Laboratory. Data was collected on pre designed questionnaire for age and anthropometric measurement for waist and hip (in centimeters), Acanthosis nigricans, fasting blood sugar, fasting serum insulin levels and insulin resistance. Insulin resistance (IR) was calculated by fasting glucose: insulin ratio (G:I). G:I <or= 4.5) was cut off for diagnosis of IR.

The data were subjected to Statistical Package for Social Sciences (SPSS) version 16 for frequency percentage, means, association and correlation of variables.

## RESULTS

 Fifty patients were included in the study with the clinical features or sonographic findings of PCO. All these women were scrutinized by a careful history and examination. Two women were diagnosed to be pregnant and one case each of hypothyroidism and hyperprolectinemia were excluded from the study. Using the Rotterdam criteria 46 (92%) women were diagnosed to have polycystic ovary syndrome and were selected for study.

According to age distribution of patients, 13 (28.3%) were less than 20 years, and 33 (71.7%) above 20 years of age. The prevalence of IR was 34.80%. Frequency of other variables was found to be as follows.

**Fig.1:**
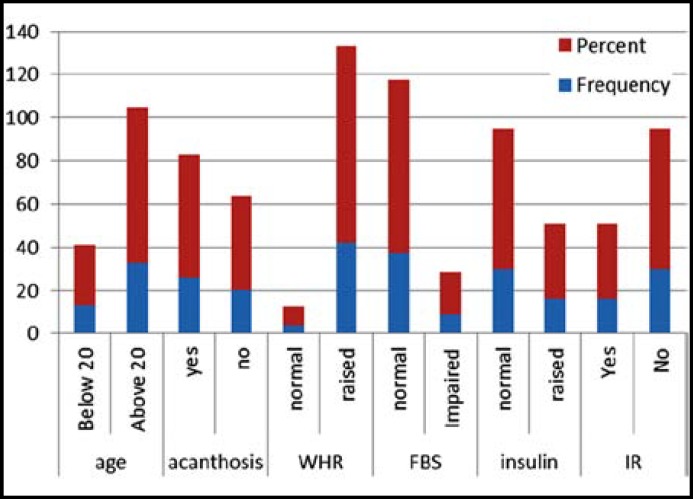
Frequency distribution of clinical signs in PCOS

Acanthosis 26(56.5%), raised WHR 42(91.3%), normal fasting blood sugar 37(80.4%), impaired fasting blood sugar 9(19.6%), raised fasting insulin was 16(34.8%) (Fig.1). Our results revealed that, the mean age of the patients was 23.72 years similarly mean of Waist- Hip Ratio (WHR), fasting blood sugar and insulin resistance was 80.89, 91.26, 0.88, 89.09 and 11.17 respectively (Table-I).

**Table-I:** Descriptive Statistics of PCOS. (n=46

	*Mean*	*Std. Deviation*
Age (yrs.)	24	4.370
Waist (cms)	80.89	17.27
Hip (cms)	91.26	16.65
WHR	0.88	0.07
Fasting Blood Sugar (mg/d)	89.09	11.63
Fasting Serum Insulin	13.70	9.17
IR	11.17	9.85

**Table-II:** Comparison of Acanthosis, with other parameters of Insulin resistance

	*Acanthosis*	*N*	*Mean*	*Std. Deviation*	*t*	*p-value*
Fasting Blood Sugar	Yes	26	93.00	11.20	2.791	0.008**
	No	20	84.00	10.34		
Fasting Serum Insulin	Yes	26	15.69	10.45	1.715	0.093
	No	20	11.10	6.57		
IR	Yes	26	10.54	10.10	-0.486	0.630
	No	20	11.97	9.70		
WHR	Yes	26	0.90	.075	2.076	0.044*
	No	20	0.86	.047		

**Highly significant at p<.01

*Significant at p< .05

**Table-III:** Pearson Correlation between different variables

	*Waist*	*HIP*	*WHR*	*Fasting Blood Sugar*	*Fasting Serum Insulin*	*IR*
Waist	1	.930^**^	.595^**^	.322^*^	.144	.023
Hip		1	.264	.201	.117	.044
WHR			1	.378^**^	.084	-.001
Fasting Blood Sugar				1	.244	.189
Fasting Serum Insulin					1	-.723^**^
IR						1

**Highly significant at p<.01

*Significant at p<.05

The Independent sample t-test was applied for the mean significant difference between the Acanthosis with fasting blood sugar, WHR, and Fasting Serum Insulin and IR. The mean significance difference was found in fasting blood sugar and WHR. The mean significant difference of fasting blood sugar was found to be positive, indicating that the patients with acanthosis have high mean value (93.0 + 11.2) as compared to the patients without acanthosis (84+10.34). (P=0.008) and show a very close variation (Table-II).

Correlation between variables showed a strong positive correlation between waist & hip ratio (r=0.93), they are significantly correlated with each other, similarly Waist & WHR shows positive correlation(r=0.59), highly significantly at p< .01. Significant positive correlation was also found between waist and FBS (r=0.32) and FBS & WHR (r=0.378) as shown in (Table-III).

## DISCUSSION

Insulin resistance is a condition in which a given concentration of insulin produces a less than expected biological effect. The prevalence of Insulin Resistance (IR) in our sample population was found to be 34.78%. In a study from Baghdad (Iraq) prevalence of insulin resistance was 76.5% however insulin resistance was calculated by HOMA (Homeostasis model assessment) test and most of the women were obese.^15^ IR was reported 52.8% in North Indian^16^ and 31% in Pakistani women with PCOS.^17^

Another study conducted on Japanese women showed insulin resistance in 52.9% of women, this result was also not consistent with our study, as insulin resistance was calculated by another method and patients in study were lean.^18^ In a study from Pakistan the mean age of woman with PCOS was reported 27.1 ± 33.5 3 which is close to our study.^19^

Though a woman may be genetically predisposed to developing PCOS, it is only the interaction of environmental factor (obesity) with the genetic factors that results in clinical expression of the PCOS. In our study raised WHR was found in 91.30% patients with the mean of 0.88± 0.70. A study from Finland reported mean Waist, Hip ratio of 0.86± 0.01 in women with PCOS.^20^ Women with PCOS showed increased prevalence of overweight, obesity and central obesity compared with women without PCOS.^21^

A Korean study showed that 61% women with PCOS were lean, 10.3% were overweight and 28.4% were obese.^22^ Obesity is 75% in women from Alabama, Birmingham.^23^ In Thai women the prevalence of insulin resistance is 20% and impaired fasting glucose is 3.2%, similar to our results 19.6%, however, prevalence was lower as population was young and thin.^24^ Cutoff point for insulin resistance was found to be different in different ethnic groups and this differences could be due to race and ethnicity.

Impaired fasting glucose was found in 12.30% PCO women in Czech Republic.^12^ The frequency of acanthosis nigricans was 56.50% in the present study. Prevalence of acanthosis was lower in Thai women which were 15.7%, but prevalence of insulin resistance was 30.7% which is very much similar to our study.^25^

Study from Institute of Endocrinology Karachi documented mean insulin level of 19.59± 14.62 and hyperinsulinemia of 75.32% in women with PCOS,^17^ these findings were close to our study. Strength of this study is that it provides local data on IR in PCOS cases. Limitation is small sample size and need for using more advanced methods for evaluating insulin resistance.

## CONCLUSION

Central obesity, acanthosis nigrican, raised WHR and FBS are significant determinant for insulin resistance in cases of PCOS.

## RECOMMANDATIONS

Clinical examination for WHR and acanthosis nigrican is strongly recommended in cases of PCOS to identify women at risk for IR and its associated complications.

## Authors contribution

Dr. Rumina Tabassum: substantial contributions to conception and design, funding, acquisition, analysis and interpretation of data, drafting the article and revising it critically for important intellectual content and final approval of the version to be published.

Dr. Fouzia Imtiaz: data collection, revision of manuscript and data interpretation and final approval of the version to be published.

Dr. Shaheen Sharafat: data interpretation, review and final approval of the manuscript.

Dr. Shazia Shukar-ud-din and Dr. Uzma Nusrat: draft preparation, data collection and final approval of the manuscript.
